# Poor quality data challenges conclusion and decision making: timely analysis of measles confirmed and suspected cases line list in Southern Nations Nationalities and People’s Region, Ethiopia

**DOI:** 10.1186/s12879-018-2983-2

**Published:** 2018-02-12

**Authors:** Misganu Endriyas, Tarekegn Solomon, Bekele Belayhun, Emebet Mekonnen

**Affiliations:** 1 Health research and technology transfer support process, Southern nations nationalities and people’s regional health bureau, Hawassa, Ethiopia; 2 College of Medicine and Health Sciences, School of Public and Environmental Health, Hawassa University, Hawassa, Ethiopia; 3 Ethiopian Public Health Association, Addis Ababa, Ethiopia

**Keywords:** Surveillance, Vaccination status, Measles, SNNPR, Healthcare seeking

## Abstract

**Background:**

Measles is one of the leading causes of death among young children even though a safe and cost-effective vaccine is available. Timely analysis of measles surveillance data is crucial for epidemic control and can show disease control program status. Therefore, this study aimed to show vaccination status and delay in seeking health care using surveillance data.

**Methods:**

A retrospective study was carried out in Southern Nations Nationalities and People’s Region (SNNPR), Ethiopia. We reviewed 2132 records from measles surveillance line list data from July 2013 to January 2014. Descriptive statistics were performed using SPSS 20 for Windows.

**Results:**

From a total of 2132 confirmed and suspected measles cases, 1319 (61.9%), had at least one dose of measles containing vaccine; the rest 398 (18.7%) and 415 (19.5%) were unvaccinated and had unknown status respectively. About two fifth, 846 (39.7%), cases visited health facilities within 48 h of onset of clinical signs/symptoms with a median of 2.0 days, IQR (1.0, 3.0).

**Conclusion:**

Majority of the measles cases were vaccinated with at least one dose of measles containing vaccine and vaccination data or vaccine potency at lower level was unclear. Delay in seeking healthcare was noted as only about two fifth of cases visited health facilities within 48 h of clinical manifestation. Vaccination and surveillance data quality and factors associated with delay in seeking health care should be investigated.

## Background

Measles is acute viral illness caused by a virus in the family paramyxovirus, genus Morbillivirus [1, 2]. Though measles is usually a mild or moderately severe illness, it can result in complications such as pneumonia, encephalitis and death. Post infectious encephalitis may occur in approximately one per 1000 reported measles cases. Approximately two to three deaths may occur for every 1000 reported measles cases [1].

Measles is one of the leading causes of death among young children even though a safe and cost-effective vaccine is available [3, 4]. Globally, measles death was 145,700 in 2013 [3] and 114,900 in 2014 [4].

In Ethiopia, estimated measles incidence was 6.52 per 100,000 populations in 2013 and 14.61 in 2014 [5]. In Southern Nations Nationalities and People’s Region (SNNPR), measles was the 6th cause of under-five admission in 2013/14 [6].

Countries in measles mortality reduction phase (areas where measles is endemic) are advised to use the clinical classification scheme until their program meet the low levels of measles incidence (measles incidence to less than five cases per million population [7]) and access to proficient measles laboratory that is access to standardized quality-controlled testing through the WHO Measles and Rubella Laboratory Network. Laboratory confirmation may be attempted by sampling approximately 10 cases per outbreak. When measles is endemic, routine monthly reporting of aggregated data on clinical measles cases is recommended by district, age group and immunization status; that is only outbreaks (not each case) are investigated [8].

In SNNPR, before 2015, samples from suspected measles cases were collected and sent to central (national) laboratory for confirmation. The occurrence of 3 or more confirmed cases within one month in a district was considered an outbreak per national guideline [9]. After outbreak confirmation, appropriate actions (like vitamin A supplementation, supplementary vaccination and severe case management) were taken based on surveillance data and risk assessments [9, 10].

Measles surveillance is critical strategy to control measles outbreak. It helps to detect outbreaks early, provide trends in transmission, and can provide incidence estimates [11]. So, this study aimed to show vaccination status and delay in seeking health care using surveillance data.

## Methods

**Fig. 1:**
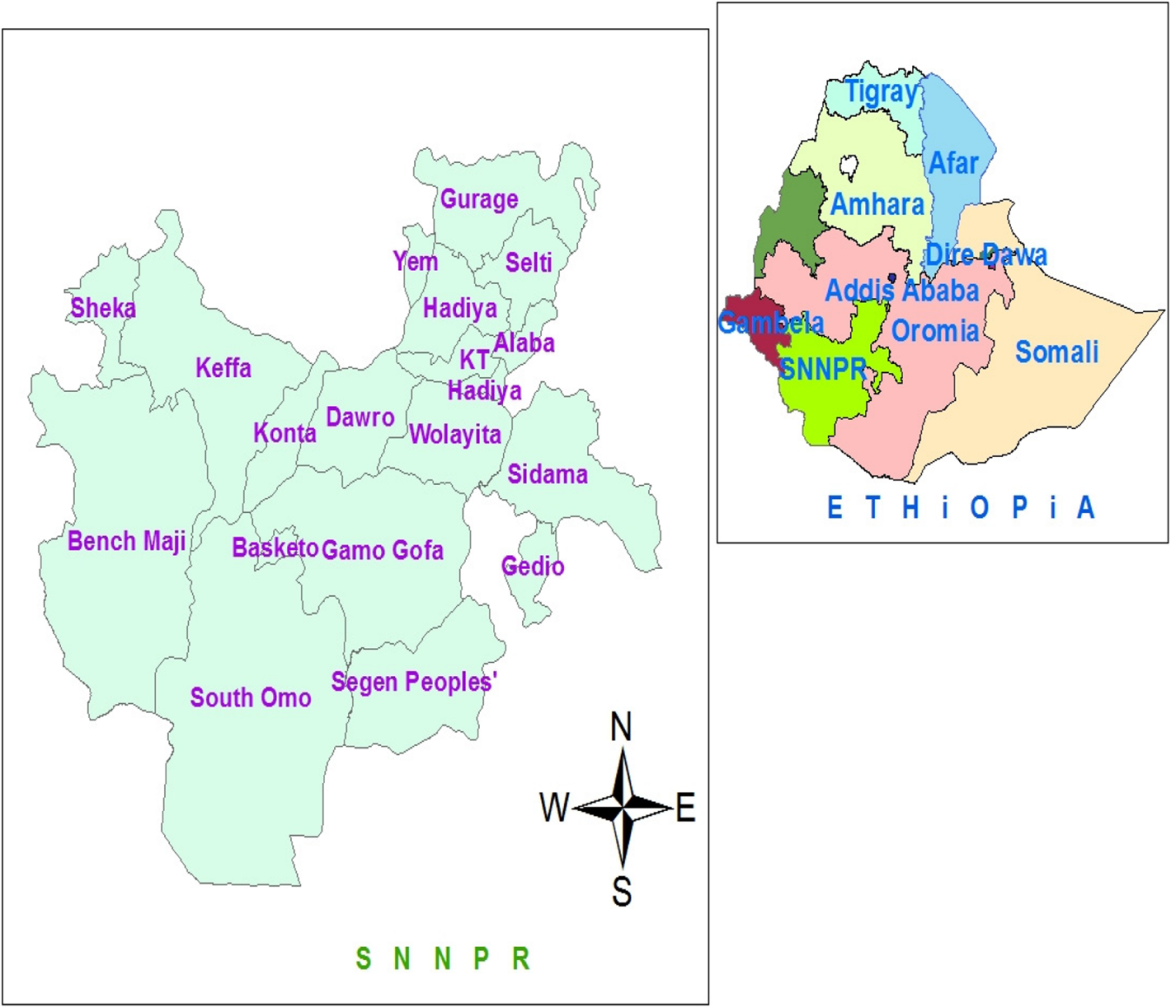
Administrative map SNNPR

A retrospective study was conducted in SNNPR using secondary data from measles surveillance line lists in September 2014. SNNPR is the third largest administrative region of Ethiopia representing about 20% of the country’s population (Fig. 1). According to 2007 census, the regional population was estimated to be 18.9 million in 2014. It is the most diverse region in the country in terms of language, culture and ethnic background. From total population, under 1 year of age was estimated to be 3.19% while less than 5 years constituted 15.6% respectively. Administratively the region is divided into 14 zones, 1 city administration and 4 special woredas (districts) [12]. Woreda, equivalent to district, is administrative structure in the region with about 100,000 populations.

A total of 2132 sample was selected using simple random sampling from suspected and confirmed measles cases reported to SNNPR health bureau from July 2013 to January 2014. The sample size was calculated using single population formula assuming proportion of cases vaccinated *P* = 0.492 [13], Zα/_2_ = 1.96 reliability coefficient for 95% confidence interval for normal distribution, margin of error d = 0.03 and design effect of 2 considering vaccination coverage variability across the region. Cases selected with incomplete and/or invalid record were replaced with next case with complete information.

### Case definition

Before 2015, the study region had limited access to proficient measles laboratory and samples from suspected cases were tested at central (national) laboratory to confirm outbreak and the region used the clinical classification scheme. In a district of about 100,000 population, measles outbreak was considered when 3 or more cases were confirmed within a month as per national guideline [9]. Laboratory investigation was done for the first 5–10 cases to confirm an outbreak in each district. After confirming an outbreak, cases that were linked to confirmed cases within a district were considered as epidemiologically confirmed. Cases from districts where rubella outbreak was confirmed were not included in this study since vaccine in the region was measles monovalent (did not include rubella and mumps).

Variables on vaccination status, age, sex, treatment modality (inpatient/outpatient), date of onset of rash, date seen at facilities, diagnosis and outcome (live or dead) were collected using checklist prepared for this purpose. During surveillance, vaccination status was collected using vaccination card, vaccination register and/or history of vaccination by health worker reporting cases.

Data was checked for its completeness and consistency and entered to and analysed using IBM SPSS for Windows version 20. Frequencies, percentages, median and interquartile were carried out.

Ethical clearance was obtained from Ethical Review Committee of SNNPR Health Bureau. No name of case was mentioned and the data were only used for the above mentioned objectives and kept confidential.

## Results

A total of 2132 confirmed and suspected measles cases were selected from cases reported to SNNP regional health bureau from July 2013 to January 2014 and reviewed for vaccination status and delay in seeking health care (time interval from date of clinical signs and symptoms to date seen at health facilities for health care).

From the total 2132 sample, 94 (4.4%) were confirmed by laboratory test while rest were epidemiologically linked. Both sexes were affected equally with male to female sex ratio of 1:1 and more than half, 1204 (56.47%), of cases were between age range of 5–14 years with a median of 8.00 (IQR 3.0, 12.0) (Table 1).

**Table 1:** Age, sex and treatment modality of measles confirmed and suspected in SNNPR, Ethiopia, 2014

Variables	Treatment modality		Total Number (%)
	Inpatient^a^	Outpatient	
	Number (%)	Number (%)	
Sex			
Female	170 (15.9%)	901 (84.1%)	1071(50.2)
Male	175 (16.5%)	886 (83.5%)	1061(49.8)
Age category			
< 1	53 (18.0%)	242 (82.0%)	295 (13.8)
1–4	79 (18.4%)	350 (81.6%)	429 (20.1)
5–14	163 (13.5%)	1041 (86.5%)	1204 (56.5)
15+	50 (24.5%)	154 (75.5%)	204 (9.6)
Total	345 (16.2%)	1787 (83.8%)	2132 (100.0)

^a^Inpatient - Patients admitted in health facility and followed for management of complication/s with fluid, vitamin A and antibiotics for at least one overnight stay

Majority, 1787 (83.8%), of cases were not admitted to heath facility or treated in outpatient department. Regarding treatment outcome, only one death was reported. The admission rate in both sex was also nearly equal with sex ratio of (male to females) 1.03 (Table 1).

More than half, 1319 (61.9%), of cases were vaccinated with at least one dose of measles containing vaccine from which 49.5% were female. About one forth, 535 (25.1%), of cases were vaccinated with two or more doses of measles containing vaccine through routine and/or supplementary vaccination campaigns. About one fifth, 398 (18.7%), of cases were unvaccinated while vaccination status of 415 (19.5%) cases was unknown (Table 2).

**Table 2:** Vaccination status of measles confirmed and suspected cases in SNNPR, Ethiopia, 2014

Variable	Dose			
	Unvaccinated	1	2+	Unknown
Age(years)				
< 1	140 (47.5%)	121 (41.0%)	24 (8.1%)	10 (3.4%)
1–4	32 (7.5%)	155 (36.1%)	216 (50.3%)	26 (6.1%)
5–14	163 (13.5%)	480 (39.9%)	292 (24.3%)	269 (22.3%)
≥ 15	63 (30.9%)	28 (13.7%)	3 (1.5%)	110 (53.9%)
Sex				
Female	196 (18.3%)	384 (35.9%)	269 (25.1%)	222 (20.7%)
Male	202 (19.0%)	400 (37.7%)	266 (25.1%)	193 (18.2%)
Treatment modality				
Inpatient	78 (22.6%)	136 (39.4%)	68 (19.7%)	63 (18.3%)
Outpatient	320 (17.9%)	648 (36.3%)	467 (26.1%)	352 (19.7%)
Total	398 (18.7%)	784 (36.8%)	535 (25.1%)	415 (19.5%)

About two fifth, 846 (39.7%), cases visited health facilities within 48 h of onset of sign and symptom. Health care seeking behavior among male and female was almost equal; 433 (40.4%) male and 413 (38.9%) female visited health facility within 48 h of onset of illness. From those who visited health facility within 48 h, 120 (14.2%) were admitted while 225 (17.5%) were admitted from those who visited health facility after 48 h. The median delay time was 2.0 days (IQR 1.0, 3.0) (Table 3).

**Table 3:** Delay in seeking healthcare of measles confirmed and suspected cases in SNNPR, Ethiopia, 2014

Variable	Time interval to visit health facilities		
	Within 48 h.	2–3 day/s	4+ days
Age (years)			
< 1	118 (40.0%)	132 (44.7%)	45 (15.3%)
1–4	150 (35.0%)	210 (49.0%)	69 (16.0%)
5–14	504 (41.9%)	538 (44.7%)	162 (13.4%)
15–44	74 (36.3%)	99 (48.5%)	31 (15.2%)
Vaccination status			
Unvaccinated	169 (42.5%)	155 (38.9%)	74 (18.6%)
One dose	347 (44.3%)	364 (46.4%)	73 (9.3%)
≥ 2 dose	183 (34.2%)	262 (49.0%)	90 (16.8%)
Unknown	147 (35.4%)	198 (47.7%)	70 (16.9%)
Sex			
Female	433 (40.4%)	487 (45.5%)	151 (14.1%)
Male	413 (38.9%)	492 (46.4%)	156 (14.7%)
Treatment modality			
Inpatient	120 (34.8%)	167 (48.4%)	58 (16.8%)
Outpatient	726 (40.6%)	812 (45.5%)	249 (13.9%)
Total	846 (39.7%)	979 (45.9%)	307 (14.4%)

## Discussion

About three fifth, 1319 (61.9%), of cases were vaccinated with at least one dose of measles containing vaccine while 398 (18.7%) and 415 (19.5%) cases were unvaccinated and had unknown status respectively.

The vaccination schedule for measles in the country is nine months (child is expected to get vaccination before age of 1 year). At community level, routine vaccination of single dose is given. Nationally, mass campaign of measles vaccination as catch-up was conducted from 2002 to 2004 targeting 6 months to 14 years. Following that, from 2005 to 2009, follow-up campaign was conducted targeting 9 months to 4 years. And from 2010 to 2011, another follow-up supplementary immunization activities (SIAs) were conducted in two phases. The first phase (2010) was conducted in SNNPR while second done in other regions that implemented their last follow up SIAs in 2009 [14]. Following increased incidence of measles, measles SIA was integrated with polio campaign and targeted children between 6 months to 15 years of age. In most cases, if there is measles outbreak, supplementary vaccination can be given. In addition, if there is case build (increments of cases over time but below outbreak threshold) and early warning situations of outbreak like malnutrition, supplementary vaccination can be given. So, there is a chance of getting more than one dose and that was why some cases got two and more doses.

Analysis of different measles outbreak surveillance data showed that outbreak can occur in area where there is high vaccination coverage (≥95%) due to accumulation of unvaccinated individuals over time and/or immigrants from low vaccination coverage [15–17]. Most epidemiological analysis of measles surveillance data also showed that the status of more than half of cases was unvaccinated [17–21]. The proportion of vaccinated cases in this study was found to be higher (61.9%) than that of epidemiological analysis of measles surveillance data done in Cameron [13], Iran [22], Iraq [21] and Italy [18] that showed 49.2%, 20%, 18% and 5.5% of cases were vaccinated respectively. At the time of outbreak, it was concluded that outbreak was due to accumulation of susceptible population. But surveillance data showed that more than half were vaccinated with at least one MCV. This higher vaccination status report could be false report of vaccination or poor efficacy of vaccine at lower level due to different reasons like cold chain failure. Proportion of cases with unknown status was also higher than other epidemiological analysis of measles outbreak [18, 21] which might indicate poor documentation of vaccination data. Ruling out data quality or cold-chain problem thus needs other study.

To control the measles outbreak, timely measles surveillance is one of critical strategies [11]. Prompt recognition, reporting, and investigation of measles is important because the spread of the disease can be limited with early case identification and public health response including vaccination. Regular monitoring of surveillance indicators, including time intervals between diagnosis and reporting and completeness of reporting, may identify specific areas of the surveillance and reporting system that need improvement. The median interval between rash onset and notification of a public health authority is one of surveillance indicators that should be monitored [1]. But, in most cases, the number of reported cases of measles reflects a small proportion of the true number of cases occurring in the community [23]. Many measles cases do not seek health care or, if diagnosed, are not reported [11] while some cases go to traditional healers as seen in Bayelsa, Nigeria [24]. At the time of measles outbreak, surveillance is highly affected by community health seeking behavior and belief, especially mothers [24, 25]. In this study, median (delay time in days) interval between rash onset and case seen in health facilities seeking health care was 2.0 (IQR 1.0, 3.0). About two fifth cases, 846 (39.7%), visited health facilities within 48 h of onset of clinical sign and symptoms (Table 3) which was lower than that of Cameroon which reported 48.5% of cases visited within 48 h of onset of clinical sign and symptoms [13].

According to WHO guideline for epidemic preparedness and response to measles outbreaks, measles is one of the most highly communicable diseases in man, with a basic reproductive rate of 17–20. The disease is communicable from slightly before the prodromal period to four days after the appearance of the rash [26]. When cases visit health facilities in time, in addition to counselling on supportive care, vitamin A is given to all cases irrespective of whether it has previously been administered for prophylaxis or given during routine immunization activities since vitamin A minimizes complication (mortality) related to measles [4]. So, cases that delayed in the community were source for disease spread and challenge outbreak control. In addition, cases that stay without care were at risk of complications related to measles. Beyond this, as this study used secondary data, nothing was known about those cases who did not visit health facility which spread the disease and expected to be at risk of developing complications.

This study was limited in assessing reasons for delay as it used secondary data. In addition, the vaccination status was also judged by health worker reporting cases using immunization card, register and/or history.

## Conclusions

Majority of the measles cases were vaccinated with at least one dose of measles containing vaccine and vaccination data or vaccine potency at lower level was unclear. Delay in seeking healthcare was noted as only about two fifth of cases visited health facilities within 48 h of clinical manifestation. Vaccination and surveillance data quality and factors associated with delay in seeking health care should be investigated.

## Abbreviations

E.F.Y: Ethiopian Fiscal Year
IBM: International Business Machines Corporation
IQR: Interquartile
PHEM: Public Health Emergency Management
SIAs: Supplementary Immunization Activities
SNNP: Southern Nations, Nationalities, and People’s
SNNPR: Southern Nations, Nationalities, and People’s Region
SPSS: Statistical Package for the Social Sciences
WHO: World Health Organization

## References

[1] Preeta Kutty, Jennifer Rota, William Bellini, Susan B. Redd, Albert Barskey, Wallace G. Vaccine-Preventable Diseases Surveillance Manual: Measles: CDC; 2013. Available from: http://www.cdc.gov/vaccines/pubs/surv-manual/chpt07-measles.pdf.

[2] Immunization, Vaccines and Biologicals: Measles: WHO; 2013 [Aug 12, 2014]. Available from: http://www.who.int/immunization/diseases/measles/en/.

[3] WHO. WHO warns that progress towards eliminating measles has stalled. Geneva: WHO media center; 2014. [cited 2016 May 10]. Available from: http://www.who.int/mediacentre/news/releases/2014/eliminating-measles/en/.

[4] WHO. Measles: Fact sheets: WHO; Media center [updated March 2016; cited 2016 May 10]. Available from: http://who.int/mediacentre/factsheets/fs286/en/.

[5] WHO. Reported measles cases and incidence rates by WHO Member States 2013 and 2014. 2015.

[6] RHB. Annual performance review meeting of 2006 EFY (2013/14). Hawassa: Regional Health Bureau, 2014.

[7] WHO. Global immunization vision and strategy: Progress report and strategic direction for the Decade of Vaccines. World Health Organization, 2011.

[8] WHO. WHO–recommended standards for surveillance of selected vaccine-preventable diseases. Measles. Geneva: WHO: Department of Vaccines and Biologicals; 2003.

[9] EHNRI (2012). Guideline on measles surveillance and outbreak management.

[10] WHO. Response to measles outbreaks in measles mortality reduction settings. Geneva: WHO; 2009. 23785742

[11] WHO. World malaria report 2014. World Health Organization, 2014.

[12] RHB. SNNP Region Overview: Regional Health Bureau; [cited 2014 Aug 13]. Available from: http://www.snnprhb.gov.et/index.php?option=com_content&view=article&id=9:sexteneded&catid=2:vision-mission&Itemid=39.

[13] Sume GE, Kobela M, Delissaint D, Kazambu D, Emah I (2014). Case based measles surveillance performance in 2010, littoral region of Cameroon. J Publ Health in Afr.

[14] Shemsedin Toyba, Sisay Gashu, Fiona Braka, Mitike Molla, Masresha B. Implementing Best Practice Measles SIAs: The Ethiopia Experience. 2011.

[15] Minetti A, Kagoli M, Katsulukuta A, Huerga H, Featherstone A, Chiotcha H (2013). Lessons and challenges for measles control from unexpected large outbreak, Malawi. Emerg Infect Dis.

[16] Nkowane BM, Bart SW, Wao BM (1987). Measles outbreak in a vaccinated school population: epidemiology, chains of transmission and the role of vaccine failures. Am J Public Health.

[17] Dominguez A, Torner N, Barrabeig I, Rovira A, Rius C, Cayla J (2008). Large outbreak of measles in a community with high vaccination coverage: implications for the vaccination schedule. Clin infectious dis: an official publication of the Infec Dis Soc Am.

[18] Filia A, Bella A, Rota M, Tavilla A, Magurano F, Baggieri M (2013). Analysis of national measles surveillance data in Italy from October 2010 to December 2011 and priorities for reaching the 2015 measles elimination goal. Euro Surveill..

[19] Delaporte E, Wyler Lazarevic CA, Iten A, Large SP. Measles outbreak in Geneva, Switzerland, January to august 2011: descriptive epidemiology and demonstration of quarantine effectiveness. Euro Surveill. 2013;18(6) 23410259

[20] Hyde TB, Dayan GH, Langidrik JR, Nandy R, Edwards R, Briand K (2006). Measles outbreak in the Republic of the Marshall Islands, 2003. Int J Epidemiol.

[21] Jasem J, Marof K, Nawar A, Monirul Islam KM (2012). Epidemiological analysis of measles and evaluation of measles surveillance system performance in Iraq, 2005-2010. Int J infectious dis: IJID : official publication of the Int Soc Infectious Dis.

[22] Salimi V, Abbasi S, Zahraei SM, Fatemi-Nasab G, Adjaminezhad-Fard F, Shadab A (2014). Implementation of a national measles elimination program in Iran: phylogenetic analysis of measles virus strains isolated during 2010-2012 outbreaks. PLoS One.

[23] Jani JV, Jani IV, Araújo C, Sahay S, JB BG. Assessment of routine surveillancedata as a tool to investigate measles outbreaks in Mozambique. BMC Infect Dis. 2006;6(29) 10.1186/1471-2334-6-29 PMC1388222 16504049

[24] Adika VO, Baralate S, JJA NN (2013). Mothers perceived cause and health seeking behaviour of childhood measles in Bayelsa, Nigeria. J Res Nursing and Midwifery (JRNM).

[25] Lim T-A, Marinova L, Kojouharova M, Tsolova S, Semenza JC (2013). Measles outbreak in Bulgaria: poor maternal educational attainment as a risk factor for medical complications. Eur J Pub Health.

[26] WHO. Guidelines for Epidemic Preparedness and Response to Measles Outbreaks. Geneva, Switzerland: WHO; 1999.

